# Use and self-perceived effects of social media before and after the COVID-19 outbreak: a cross-national study

**DOI:** 10.1007/s12553-021-00595-x

**Published:** 2021-09-14

**Authors:** Hilde Thygesen, Tore Bonsaksen, Mariyana Schoultz, Mary Ruffolo, Janni Leung, Daicia Price, Amy Østertun Geirdal

**Affiliations:** 1 Faculty of Health Sciences, Department of Occupational Therapy, Prosthetics and Orthotics, Oslo Metropolitan University, Oslo, Norway; 2 Faculty of Health Studies, VID Specialized University, Oslo, Norway; 3 Faculty of Social and Health Sciences, Department of Health and Nursing Sciences, Inland Norway University of Applied Sciences, Elverum, Norway; 4 Faculty of Health Studies, VID Specialized University, Sandnes, Norway; 5 Health and Life Sciences, Northumbria University, Newcastle upon Tyne, UK; 6 School of Social Work, University of Michigan, Ann Arbor, MI USA; 7 Faculty of Health and Behavioural Science, The University of Queensland, St. Lucia, Australia; 8 Faculty of Social Sciences, Department of Social Work, OsloMetropolitan University, Oslo, Norway

**Keywords:** Coronavirus, COVID-19, Cross-national study, Emotional distress, Mental health, Social media

## Abstract

To (i) examine the use of social media before and after the COVID-19 outbreak; (ii) examine the self-perceived impact of social media before and after the outbreak; and (iii) examine whether the self-perceived impacts of social media after the outbreak varied by levels of mental health. A cross-national online survey was conducted in Norway, UK, USA and Australia. Participants (*n* = 3810) reported which social media they used, how frequently they used them before and after the COVID-19 outbreak, and the degree to which they felt social media contributed to a range of outcomes. The participants also completed the 12-item General Health Questionnaire. The data were analyzed by chi-square tests and multiple linear regression analysis. Social media were used more frequently after the pandemic outbreak than compared to before the outbreak. Self-perceived effects from using social media increased after the COVID-19 outbreak, and in particular stress and concern for own and others’ health. Emotional distress was associated with being more affected from using social media, in particular in terms of stress and concern for own or others’ health. The use of social media has increased during the coronavirus outbreak, as well as its impacts on people. In particular, the participants reported more stress and health concerns attributed to social media use after the COVID-19 outbreak. People with poor mental health appear to be particularly vulnerable to experiencing more stress and concern related to their use of social media.

## Background

The coronavirus outbreak has caused a global health crisis. The virus, which originated in China, has spread to all continents, causing large numbers of infected and dead worldwide [1]. Due to the highly contagious nature of the virus, the World Health Organization (WHO) recommended a number of measures to prevent further spread [2]. These recommendations include limiting human-to-human transmission by reducing close contacts between people. Following these guidelines, strict rules of social distancing was established in many countries, including Norway, UK, USA and Australia. The social distancing rules implied that people should stay at home and limit any physical contact with others, outside the household, as much as possible. For people infected, or in contact with someone infected by the virus, strict quarantine rules applied. The social distancing rules caused nurseries, schools and universities to close, and a lockdown of most of society caused a number of businesses to close, resulting in unemployment rates increasing sharply [3].

Due to complying with the social distancing rules, contact with family outside the household, friends and colleagues were limited to online communication forms, which included the use of social media platforms. Social media are increasingly becoming important platforms of communication and information-exchange [4]. Social media is here understood as “computer-based technology that facilitates the sharing of ideas, thoughts, and information through the building of virtual networks and communities” [5]. In the light of the COVID-19 pandemic, social media played a vital role in connecting people, and in providing updated information about the virus [6].

The COVID-19 outbreak with its strict social distancing measures caused considerable mental health stress in the population [6–9]. This related in part to the many unpredictable aspects of the pandemic, including the growing number of patients and suspected cases across all continents, as well as the obvious concerns about getting infected. Several studies reported increased levels of anxiety and depression related to people being isolated at home, with limited possibilities for face-to-face contact during the corona outbreak [7, 9–11]. These studies point to social media as a major contributor to increased levels of stress and mental health problems, arguing that disinformation and false reports have bombarded social media during the COVID-19 outbreak, leading to misinformation overload. In a study of mental health problems and social media exposure during the COVID-19 outbreak in China, for example, the researchers found that persons spending two hours or more a day on social media reported significantly higher levels og mental health problems, and especially anxiety and depression [6]. As such, the role of social media during the corona-outbreak can be understood as ambiguous, as on the one hand it enabled people to stay in touch and be updated on the situation, while at the same time contributing to substantial stress and worry. For example, research has shown that social media may create a sense of community and connectedness [12] and increase one’s social capital [13], but also that it may increase symptoms of anxiety and depression [10, 14].

Despite the growing literature on social media, comprehensive comparative studies of social media use, their perceived effects on individuals and their relationship to mental health are scarce. The COVID-19 outbreak constitutes extraordinary circumstances under which whole populations are subject to restrictions and stress. While the use of social media can help to relieve some of that stress by allowing for social contact without physical proximity, negative effects, such as increased worry, are equally viable. Moreover, it is possible that differences in mental health can contribute to determine the perceived impact of social media use. If this is the case, the study may have implications for the approach taken to social media use for different population subgroups.

## Study aims

The aims of this study were to (i) examine the use of social media before and after the COVID-19 outbreak; (ii) examine the self-perceived impact of social media before and after the outbreak; and (iii) examine whether the self-perceived impacts of social media after the outbreak varied by levels of mental health while adjusting by sociodemographic variables.

## Methods

In April 2020, the general population from Norway, USA, UK and Australia was invited to participate in the cross-sectional study through a self-administered survey. The online survey was distributed via different social media such as Facebook, Instagram and Twitter. The sampling in each country was done by convenience, implying that all participants opted to participate by self-selection. Each country had a survey landing site at the researcher’s universities; OsloMet—Oslo Metropolitan University, Norway; University of Michigan, USA; University of Salford, UK; and the University of Queensland, Australia, respectively. AØG from OsloMet conceptualized and initiated the project. All countries and universities had their own project lead in order to comply with the ethical considerations and permissions of the country and/or institution. Each project lead was able to speak the language of the landing site and English. To ensure that participants were able to understand the survey questions, the survey was presented in Norwegian (to the participants from Norway) and in English (to the participants from USA, UK, an Australia).The data was collected in April and May 2020.

### Participants

Participants had to be 18 years or older. They had to understand Norwegian or English and live in Norway, USA, UK or Australia.

### Measures

#### Sociodemographic characteristics

Sociodemographic variables included age group (18–29 years, 30–39 years, 40–49 years, 50–59 years, 60–69 years, 70 years and older), sex (male, female, not stated or other), living area (rural/farming area, small town, medium sized city, large city), highest completed education level (elementary school, high school, associated/technical degree, bachelor’s degree, master’s degree/doctoral degree), cohabitation (living with someone else or not), and employment status (being in full-time or part-time employment versus being unemployed).

#### Social media use

The participants were asked to indicate whether they had used any of the nine listed well known social media channels before the COVID-19 outbreak. The list was based on US statistics of the most common social media platforms in 2019, and included the following: Facebook, YouTube, Snapchat, Instagram, LinkedIn, Twitter, Pinterest, WhatsApp and Reddit [15]. The participants were then asked how often they had used social media in general (i.e., not for each type of social media) before and after the COVID-19 outbreak. Data were collected at one time-point after the COVID-19 outbreak, therefore, “before” was retrospectively recalled by participants. Response options for these items were monthly or less frequently = 1, weekly = 2, a few times per week = 3, daily = 4, or several times per day = 5.

The participants were then asked to indicate the self-perceived effects of using social media before and after the COVID-19 outbreak. Listed effects were support, communication, stress, information, being updated, concern for own or others’ health, engagement, relaxation, and ‘other’. For each of the listed effects, the participants had option to indicate if the use of social media: had no contribution = 1, contributed a little = 2, contributed somewhat = 3, or contributed much = 4 to the listed effects.

#### Mental health

General Health Questionnaire 12 (GHQ-12) is widely used as a self-reported measure of mental health [16, 17]. A large number of studies in the general adult-, clinical- work and student population have provided support for its validity and reliability across various samples and contexts [17–22]. Six items of the GHQ-12 are phrased positively (e.g. ‘able to enjoy day-to-day activities’), while six items are phrased as negative experiences (e.g. ‘felt constantly under strain’). On each item, the person indicates the degree to which the item content has been experienced during the two preceding weeks, using four response categories (‘less than usual’, ‘as usual’, ‘more than usual’ or ‘much more than usual’). Items are scored between 0 and 3, and positively formulated items are recoded prior to analysis. As a result, the GHQ-12 scale score range is 0–36, with higher scores indicating poorer mental health (more psychological distress). If the person responded ‘more than usual’ or ‘much more than usual’ on at least four of the 12 items, this indicates a level of emotional distress where treatment may be needed (‘case-level score’) [23]. In this study, participants with and without case-level scores were compared with regards to the effects they perceived from using social media.

### Statistical analysis

The whole sample, and each of the national subsamples, were described with frequencies and percentages for categorical variables and means and standard deviations for continuous variables. Overall differences in proportions between groups were analyzed with the Chi-Square test, whereas overall group differences in mean scores were analyzed with the *F*-test of the one-way analyses of variance (ANOVA). Differences in self-perceived impact of social media before and after the COVID-19 outbreak were analyzed with the dependent *t*-test.

Associations with self-perceived effects from using social media after the COVID-19 outbreak were investigated with linear regression analysis. Scores on support, communication, stress, information, be updated, concern, engagement and relaxation were used as dependent variables in eight subsequent regression analyses. All analyses included age group, gender, education level, and employment as potential confounders and emotional distress as the independent variable of interest. Effects sizes were reported as standardized beta weights. Statistical significance was set at *p* < 0.05. Missing values were managed with casewise deletion, resulting in *n* varying between analyses. The software used for analysis was SPSS version 26.

### Ethics

The data in this cross-sectional and cross-country study were collected anonymously. All ethical rules were followed in each country. The study was quality assured and approved by OsloMet and the regional committees for medical and health research ethics (REK; ref. 132,066) in Norway, reviewed by the University of Michigan Institutional Review Board for Health Sciences and Behavioral Sciences (IRB HSBS) and designated as exempt (HUM00180296) in USA, by University Health Research Ethics (HSR1920-080) in UK, and (HSR1920-080) 2,020,000,956) in Australia.

## Results

### Participants

Characteristics of the sample and the sample subgroups are displayed in Table 1. Differences between countries were statistically significant for all included variables. Among the participants from Norway, 72.9% were under the age of 50 years, while the corresponding proportions were 57.0% for the UK, 52.5% for the USA and 63.4% for Australia. All countries had a solid majority of female participants (range: 74.4% in the USA to 84.4% in the UK). Between 60% (USA) and 84% (Australia) reported that they lived in an urban environment (medium-sized or large city). Higher education levels (bachelor level education or higher) were found among 67%-81% of the sample, with the lowest proportion in the UK and the highest proportion in Norway. In all countries, about 80% lived with others. Full-time employment was reported among 62.3% of the Norwegian participants, with corresponding proportions ranging 40%-48% in the other countries. Part-time employment was reported among 34% of the participants from Australia, with corresponding proportions ranging 17%-23% in the other countries. No employment was reported among 37% of the participants from the USA, with corresponding proportions ranging from 16%-30% in Norway, UK and Australia. The respondents reporting no employment were either students, retired, on maternal- or paternal leave or in working age without paid employment.

**Table 1:** Sociodemographic characteristics of the total sample and of each of the four subsamples

	Total (*n* = 3810)		Norway (*n* = 771, 20.2%)		UK (*n* = 1373, 36.0%)		USA (*n* = 1393, 36.6%)		Australia (*n* = 273, 7.2%)		
*Characteristics*	*n*	%	*n*	%	*n*	%	*n*	%	*n*	%	*p*
Age group											
18–29 years	705	18.5	188	24.4	201	14.6	241	17.4	75	27.5	< 0.001
30–39 years	713	18.7	176	22.8	236	17.2	245	17.7	56	20.5	
40–49 years	827	21.7	198	25.7	346	25.2	241	17.4	42	15.4	
50–59 years	723	19.0	116	15.0	317	23.1	243	17.5	47	17.2	
60–69 years	612	16.1	71	9.2	209	15.2	290	20.9	42	15.4	
70 years and over	224	5.9	22	2.9	64	4.7	127	9.2	11	4.0	
Sex											
Male	718	18.8	143	18.5	198	14.4	324	23.3	53	19.4	< 0.001
Female	3034	79.6	628	81.5	1159	84.4	1036	74.4	211	77.3	
Other/not stated	57	1.5	0	0.0	16	1.2	32	2.3	9	3.3	
Living area											
Rural/farming	282	7.4	63	8.2	76	5.5	138	9.9	5	1.8	< 0.001
Small town	843	22.1	150	19.5	233	17.0	421	30.2	39	14.3	
Medium sized city	1228	32.2	116	15.0	498	36.3	570	40.9	44	16.1	
Large city	1457	38.2	442	57.3	566	41.2	264	19.0	185	67.8	
Education level											
Elementary school	17	0.4	9	1.2	0.0	0.0	3	0.2	5	1.8	< 0.001
High school	380	10.0	112	14.5	127	9.2	119	8.5	22	8.1	
Assoc./techn. Degree	592	15.5	26	3.4	331	24.1	175	12.6	60	22.1	
Bachelor’s degree	1262	33.1	272	35.3	462	33.6	469	33.7	59	21.7	
Master’s/PhD degree	1558	40.9	352	45.7	453	33.0	627	45.0	126	46.3	
Cohabitation											
Yes	3079	80.8	610	79.1	1127	82.3	1115	80.2	227	83.5	0.19
No	724	19.0	161	20.9	243	17.7	275	19.8	45	16.5	
Employment											
Yes, full-time	1890	49.6	480	62.3	656	47.8	644	46.3	110	40.3	< 0.001
Yes, part-time	802	21.0	166	21.5	309	22.5	235	16.9	92	33.7	
No	1117	29.3	125	16.2	408	29.7	513	36.9	71	26.0	

Assoc./techn. degree is associate/technical degree. Statistical tests are Chi-Square tests. Cohabitation refers to ‘living with someone else’

### Use of social media

The participants’ use of various social media in the four countries are displayed in Table 2. In the whole sample, Facebook (94.6%) YouTube (69.6%) and WhatsApp (43.1%) were the most used social media. Facebook was used by a large majority of participants in each of the countries, ranging between 89% in Australia and 97% in the USA. The mean number of social media used varied significantly between countries.

**Table 2:** Use of social media in the total sample and in each of the four countries

	Total (*n* = 3810)		Norway (*n* = 771, 20.2%)		UK (*n* = 1373, 36.0%)		USA (*n* = 1393, 36.6%)		Australia (*n* = 273, 7.2%)		
*Social media*	*n*	%	*n*	%	*n*	%	*n*	%	*n*	%	*p*
Facebook^a^	3603	94.6	741	96.1	1272	92.6	1347	97.3	243	89.0	< 0.001
YouTube^a^	2652	69.6	503	67.4	842	61.3	1113	82.0	194	71.1	< 0.001
Snapchat^a^	1122	29.4	549	73.5	236	17.2	288	22.6	49	17.9	< 0.001
Instagram^a^	2121	55.7	559	75.0	672	48.9	733	56.1	157	57.5	< 0.001
LinkedIn^a^	1200	31.5	259	35.7	293	21.3	547	42.0	101	37.0	< 0.001
Twitter^a^	1194	31.3	134	18.6	480	35.0	496	38.1	84	30.8	< 0.001
Pinterest^a^	1042	27.3	172	23.8	303	22.1	513	39.5	54	19.8	< 0.001
WhatsApp^a^	1643	43.1	230	31.6	1072	78.1	221	17.2	120	44.0	< 0.001
Reddit^a^	391	10.3	41	5.8	68	5.0	252	19.7	30	11.0	< 0.001
	*M*	*SD*	*M*	*SD*	*M*	*SD*	*M*	*SD*	*M*	*SD*	*p*
Number of media used^b^	4.0	1.7	4.2	1.5	3.8	1.7	4.0	1.8	3.8	1.8	< 0.001

^a^ Statistical test is Chi-Square test; ^b^ statistical test is ANOVA *F*-test

The frequency of social media use before and after the COVID-19 outbreak is shown in Table 3. Before the COVID-19 outbreak, the proportion of all participants using social media less frequently than daily was low (ranging between 4.9% in Norway and 11.7% in Australia) and further decreased after the COVID-19 outbreak. Similar results were found about daily use. The proportion of participants using social media daily decreased substantially after the COVID-19 (between 6.3% points in Australia and 12.9% points in USA). Notably, the proportion of participants using social media several times daily increased substantially for all countries (10.7% points in Norway, 11.7% points in Australia, 17.1% points in the UK and 17.9% points in the USA).

**Table 3:** Frequency of social media use before and after the COVID-19 outbreak

	Total (*n* = 3757)			Norway (*n* = 765, 20.4%)			UK (*n* = 1356, 36.1%)			USA (*n* = 1363, 36.3%)			Australia (*n* = 273, 7.3%)		
*Social media use*	% Before	% After	*p*	% Before	% After	*p*	% Before	% After	*p*	% Before	% After	*p*	% Before	% After	*p*
Monthly or less frequent	1.1	0.4	< 0.001	0.4	0.1	< 0.001	0.9	0.7	< 0.001	1.5	0.1	< 0.001	1.8	1.1	< 0.001
Weekly	1.6	1.0		1.6	0.5		1.6	1.1		1.6	1.2		1.5	1.5	
A few times a week	5.8	2.8		2.9	2.6		7.6	3.5		5.1	2.0		8.4	3.7	
Daily	35.3	23.8		26.8	17.6		40.9	28.6		32.9	20.0		42.9	36.6	
Several times daily	56.3	72.0		68.4	79.1		49.0	66.1		58.9	76.8		45.4	57.1	

Table content is percentage (%) of the sample within each response category. Statistical tests are Chi-Square test

The frequency of social media use varied systematically by age – while using social media several times per day occurred more often than less frequent social media use in all age groups, the difference was more outspoken in the youngest age groups. Among those aged 18–29 years, 83% used social media several times daily, while the proportions ranged 53%-73% in the other age groups (*p* < 0.001). Participants with higher education were also more inclined to use social media several times daily (73%), compared to participants with lower levels of education (69%, *p* < 0.05).

### Self-perceived effects of social media use

Self-perceived effects of the participants’ use of social media before and after the COVID-19 outbreak are shown in Table 4. The three most prominent self-perceived effects of social media use were communication, keeping updated and information; these received the highest endorsement before and after the outbreak. This pattern applied to the whole sample and to each of the countries. Relaxation, as a self-perceived effect of social media use, decreased significantly from before to after the outbreak, while all other listed effects increased significantly. The largest effect sizes were found for stress (Cohen’s *d* = 0.59) and concern for own or others’ health (Cohen’s *d* = 0.66), for which participants on average reported that social media had contributed to a greater amount of these after the COVID-19 outbreak. This finding was observed in the whole sample and consistently across countries.

**Table 4:** Self-perceived effects of social media before and after the COVID-19 outbreak

*Perceived effects*	Total (n = 3810)			Norway (n = 771, 20.2%)			UK (n = 1373, 36.0%)			USA (n = 1393, 36.6%)			Australia (n = 273, 7.2%)		
	Before	After	*p*	Before	After	*p*	Before	After	*p*	Before	After	*p*	Before	After	*p*
Support ↑	2.24	2.55	< 0.001	2.15	2.55	< 0.001	2.21	2.57	< 0.001	2.29	2.51	< 0.001	2.37	2.63	< 0.001
Communication ↑	2.89	3.08	< 0.001	3.29	3.45	< 0.001	2.62	2.83	< 0.001	2.88	3.07	< 0.001	2.73	3.01	< 0.001
Stress ↑	2.02	2.59	< 0.001	1.92	2.25	< 0.001	2.06	2.67	< 0.001	2.05	2.72	< 0.001	1.88	2.45	< 0.001
Information ↑	2.84	2.99	< 0.001	3.00	3.21	< 0.001	2.73	2.79	< 0.01	2.86	3.03	< 0.001	2.87	3.04	< 0.001
Be updated ↑	2.86	3.01	< 0.001	3.06	3.22	< 0.001	2.73	2.82	< 0.001	2.88	3.05	< 0.001	2.87	3.07	< 0.001
Concern ↑	1.95	2.59	< 0.001	1.59	2.26	< 0.001	2.14	2.74	< 0.001	1.95	2.62	< 0.001	2.07	2.65	< 0.001
Engagement ↑	2.53	2.69	< 0.001	2.64	2.75	< 0.001	2.55	2.69	< 0.001	2.43	2.65	< 0.001	2.57	2.76	< 0.001
Relaxation ↓	2.39	2.19	< 0.001	2.93	2.67	< 0.001	2.27	2.07	< 0.001	2.20	2.03	< 0.001	2.32	2.14	< 0.001

Statistical tests are dependent samples t-test. ↑ increased; ↓ decreased. Mean self-perceived effects were estimated from: no contribution = 1, contributed a little = 2, contributed somewhat = 3, contributed much = 4

### Associations between emotional distress and self-perceived effects of social media use

Emotional distress was frequent in all age groups, yet more frequent among participants in the younger age groups. Among those aged 18–29 years, emotional distress was found among 79%, while ranging between 52 and 70% in the other age groups (*p* < 0.001). The proportion of participants with emotional distress did not differ by education level (66% for higher education versus 67% for lower education, *ns*.).

Table 5 displays associations between emotional distress and self-perceived effects of social media adjusted by sociodemographic variables. The included covariates (age group, gender, education level and employment status) were significantly associated with most perceived effects from using social media. However, even when adjusting by the sociodemographic covariates, emotional distress was positively associated with perceived support (*β* = 0.05, *p* < 0.01), stress (*β* = 0.30, *p* < 0.001), be updated (*β* = 0.04, *p* < 0.05), concern (*β* = 0.22, *p* < 0.001), and negatively associated with relaxation (*β* = -0.16, *p* < 0.001). The regression model explained 15.2% of the variance in perceived stress, and 7.3% of the variance in concern. Otherwise, the models had generally low predictive ability, explaining between 1.2%-4.6% of the outcome variances.

**Table 5:** Regression analyses showing associations between emotional distress and perceived effects of social media use in the whole sample after the COVID-19 outbreak

	Support	Communication	Stress	Information	Be updated	Concern	Engagement	Relaxation
*Variables*	*β*	*Β*	*β*	*β*	*β*	*β*	*β*	*β*
Age group	-0.08***	-0.18***	-0.18***	-0.12***	-0.13***	-0.08***	-0.04*	0.04*
Sex	0.12***	0.09***	0.07***	0.05**	0.04**	0.08***	0.09***	0.01
Education	0.05**	0.10***	-0.01	0.09***	0.10***	-0.05**	0.05**	0.07***
Employment	-0.05**	-0.05**	-0.02	-0.05**	-0.06**	-0.02	-0.02	-0.03
Emotional distress	0.05**	-0.01	0.30***	0.02	0.04*	0.22***	-0.01	-0.16***
**Explained variance**	2.7%***	4.6%***	15.2%***	2.4***	2.8%***	7.3%***	1.2%***	3.4%***

Variable coding: Lower age group is lower age. Male is 0, female is 1. Those reporting sex other than male/female were excluded from the analyses due to insufficient sample size. Higher education group is higher level of education. Having employment is 1, not having employment is 0. Emotional distress (indicating that at least four of the GHQ items are rated as indicating more problems than usual) is 1, not emotional distress is 0

^*^p < 0.05; **p < 0.01; ***p < 0.001

## Discussion

The aim of this study was threefold: to examine the use of social media before and after the COVID-19 outbreak; to examine the self-perceived impact of social media before and after the outbreak; and to examine whether the self-perceived impacts of social media after the outbreak varied by levels of mental health (emotional distress) while adjusted by sociodemographic variables. The study showed that from the nine listed social media investigated in the study, Facebook was the most frequently used across the four countries, while all other social media were used in different proportions among the participants from the four countries. While there were some differences in the change of use of social media between the countries, the overall change pattern was similar: the participants in all countries used social media more frequently after the pandemic outbreak when compared to before the outbreak. In addition, most of the self-perceived effects from using social media increased after the COVID-19 outbreak, of which stress and concern for own and others’ health increased the most. Self-reported emotional distress was significantly associated with a range of self-perceived effects from using social media, most notably stress and concern for own or others’ health.

Facebook was used by most participants (89%-97%) in all involved countries, and YouTube was also used by well over half (61%-82%) of the participants. These results correspond well with previous findings concerned with the popularity of various social media worldwide [24]. However, as the survey was distributed via social media channels, the use of various social media may have been more frequent among participants in this study, compared to the general population. In support of this reasoning, a recent survey found that 82% of the Norwegian general population had a personal profile on Facebook [25], well below the 97% reporting use of Facebook in this study. Moreover, the use of social media in general may increase rapidly in times of crisis, such as the ongoing COVID-19 pandemic, with increased information as well as misinformation being probable effects [10, 26, 27].

Most of the participants reported a high frequency use of social media, both before and after the COVID-19 outbreak. The biggest change in social media use, when comparing from before to after the pandemic, was the sharp increase in use of social media several times per day (from 56.3% before the pandemic to 72.0% after the onset of the pandemic). This finding may be interpreted in view of several factors. In times of crisis there is a need for critical information, which is easily accessed through social media. Also, a crisis – such as the COVID-19 pandemic—contributes to increased fear and uncertainty in the population [28]. Studies on the use of social media confirm the tendency found in our data, that the frequency of use of social media increases during times of crisis [29–31].

The participants retrospectively reported that the self-perceived effects from using social media were less outspoken before the COVID-19 outbreak, in comparison to after the pandemic. In previous research, social media have been shown to contribute to a sense of belonging and connectedness to others [32], and may thereby be an outlet for coping and support [33]. In the context of COVID-19, one study found that social media played an important buffering role by reducing anxiety during a nine-week period in the early stage of the pandemic [34]. In view of models of disaster impact over time [35], it is possible that the connection obtained by social media use may moderate the perceived impact of the crisis in its early stages. However, ways of using social media differ between users, and routine use of social media has been found to have better mental health effects compared to use characterized by emotional connection to the media [36]. The use of social media may also contribute to a way of obtaining information, which is highly relevant in the current situation as information spread via face-to-face communications have been limited due to lock-down and shelter in place policies. This is reflected in our finding that the top reasons for social media use were to keep updated, and for purposes of communication and information. Concerns have been raised that the increasingly vast and fast spread of information that have not gone through a fact-checking process may evoke public panic [26]. Future research on potential psychological distress caused by misinformation on social media may help inform communication strategies to reduce associated negative consequences.

In particular, the study has shown that the negative self-perceived effects of using social media – such as stress and concern for own or other’s health – were rated more highly after the onset of the pandemic. This is an unsurprising outcome and – to some extent – may relate to the many uncertainties introduced with the pandemic situation. These uncertainties could range from the worry about the degree of medical severity resulting from oneself or next of kin getting infected by the virus, and thereby possibly causing their deaths, to stress and concerns for the pandemic’s impact on daily tasks, work and finances. The timing of our data collection may as such have impacted on these findings. At that time (April and May 2020) the pandemic was in its relatively early stages in the included countries, as were the social distancing regulations and quarantine rules. Also, the global scale of the pandemic and the rapidly rising death tolls at the time of the survey (including the participating countries) may to some extent explain these findings.

Self-reported emotional distress was significantly associated with a range of perceived effects from using social media, most notably stress and concern for own or others’ health. Possibly, individuals with poorer mental health may be more attuned towards types of information that evoke stress and concern, as negative information may be more consistent with the more negative worldview frequently held among individuals with poor mental health. This line of reasoning is consistent with research emphasizing attentional bias in persons with mental illness [37, 38]. Individuals with higher stress levels may also be more inclined to interpret neutral or ambiguous information as threatening. Thus, attentional bias (seeking and noticing fear-consistent information) may be supplemented by an interpretive bias; that is, the tendency to interpret information in consistency with basic fear-driven assumptions. Previous research has found such interpretive bias to contribute to the understanding of individuals suffering from a range of health problems, including social anxiety and alcohol use [39], pain [40] and chronic fatigue [41]. Moreover, more increase in social media-induced stress and concern for own or others’ health among those with emotional distress may also reflect their poorer self-efficacy for managing the new demands associated with the changed situation. In support of self-efficacy impacting on stress levels, a previous Swedish study using a general population sample found that men and women with low self-efficacy were more likely to suffer from mental illness, compared to those with higher self-efficacy [42].

We found that those who were not employed were more in agreement that social media contributed to support, communication, information and to being updated. People who were not employed may include people who were no longer working due to COVID-19, people not working due to personal reasons or a disability, or people who have retired and are likely to be older. These sub-populations of our community may be placed at increased risk of vulnerability by the COVID-19 pandemic. Future research into the psychosocial consequences experienced by people who are unemployed during the pandemic is warranted to ensure well-being in this often-neglected sub-population.

### Study limitations

Whether, or to what degree, the sample is representative of the population in the four respective countries, in unknown. Representativity can be questioned—for example, the total study sample had a majority of female and urban participants. However, the age and education distributions were well in accordance with general population statistics. Nonetheless, we stress that generalization of the study results to the general population should be done with caution. Access to the internet was required to participate, but the study did not request information about consistent access to internet. Possibly, poorer access to the internet among some groups may have caused these groups to be under-represented in the study.

The study comes with several limitations due to its cross-sectional nature. Our data concerned with social media use before and after the COVID-19 outbreak were collected at the same time-point; asking participants to remember and to state in retrospect how they perceived the impacts of social media before the pandemic outbreak. This strategy makes the responses susceptible to recall and response shift biases [43]. As our study was a voluntary survey recruited through social media, we may not have captured samples of populations who may have reduced their social media use. However, due to social distancing measures resulting in populations having a heavier reliance on the use of social media to stay connected, our findings of an increased use of social media is consistent with expectations. Our results may not be used to make causal inferences. Related to this limitation, our large sample size contributed to detecting significant results that showed a small effect size. We have therefore presented the regression coefficients that are standardized, and the proportion of variances explained, to provide better data on the importance of the factors we examined.

### Conclusion

Based on the self-reports by the participants in this study, the use of social media appears to have increased during the coronavirus outbreak, as have the impacts social media have on people. In particular, the participants in this study reported more stress and health concerns attributed to social media use after the COVID-19 outbreak, compared to before. People with poor mental health appear to be particularly vulnerable to experiencing more stress and concern related to their use of social media. The study implies that social media may be fruitfully used by health authorities to disseminate relevant information to the public. However, the use of social media after the COVID-19 outbreak was perceived to add to the participants’ stress and levels of concern, and particularly so for those reporting emotional distress. Thus, for individuals with emotional distress, social media should be used with critical reflection to maximize their potential for human contact and relevant information, while at the same time minimize their potential for increased stress.

## Data Availability

The data from which the conclusions in the current study are based will be available from the corresponding author on reasonable request.
